# Effect of norepinephrine dosage and calibration frequency on accuracy of pulse contour-derived cardiac output

**DOI:** 10.1186/cc9967

**Published:** 2011-01-17

**Authors:** Matthias Gruenewald, Patrick Meybohm, Jochen Renner, Ole Broch, Amke Caliebe, Norbert Weiler, Markus Steinfath, Jens Scholz, Berthold Bein

**Affiliations:** 1 Department of Anaesthesiology and Intensive Care Medicine, University Hospital Schleswig-Holstein, Campus Kiel, Schwanenweg 21, D-24105 Kiel, Germany; 2 Institute of Medical Informatics and Statistics, Christian-Albrechts University Kiel, Arnold-Heller-Strasse 3, Haus 31, D-24105 Kiel, Germany

## Abstract

**Introduction:**

Continuous cardiac output monitoring is used for early detection of hemodynamic instability and guidance of therapy in critically ill patients. Recently, the accuracy of pulse contour-derived cardiac output (PCCO) has been questioned in different clinical situations. In this study, we examined agreement between PCCO and transcardiopulmonary thermodilution cardiac output (CO_TCP_) in critically ill patients, with special emphasis on norepinephrine (NE) administration and the time interval between calibrations.

**Methods:**

This prospective, observational study was performed with a sample of 73 patients (mean age, 63 ± 13 years) requiring invasive hemodynamic monitoring on a non-cardiac surgery intensive care unit. PCCO was recorded immediately before calibration by CO_TCP_. Bland-Altman analysis was performed on data subsets comparing agreement between PCCO and CO_TCP _according to NE dosage and the time interval between calibrations up to 24 hours. Further, central artery stiffness was calculated on the basis of the pulse pressure to stroke volume relationship.

**Results:**

A total of 330 data pairs were analyzed. For all data pairs, the mean CO_TCP _(±SD) was 8.2 ± 2.0 L/min. PCCO had a mean bias of 0.16 L/min with limits of agreement of -2.81 to 3.15 L/min (percentage error, 38%) when compared to CO_TCP_. Whereas the bias between PCCO and CO_TCP _was not significantly different between NE dosage categories or categories of time elapsed between calibrations, interchangeability (percentage error <30%) between methods was present only in the high NE dosage subgroup (≥0.1 μg/kg/min), as the percentage errors were 40%, 47% and 28% in the no NE, NE < 0.1 and NE ≥ 0.1 μg/kg/min subgroups, respectively. PCCO was not interchangeable with CO_TCP _in subgroups of different calibration intervals. The high NE dosage group showed significantly increased central artery stiffness.

**Conclusions:**

This study shows that NE dosage, but not the time interval between calibrations, has an impact on the agreement between PCCO and CO_TCP_. Only in the measurements with high NE dosage (representing the minority of measurements) was PCCO interchangeable with CO_TCP_.

## Introduction

Cardiac output (CO) monitoring in high-risk patients has gained increasing interest because early detection of hemodynamic instability can reduce morbidity in these patients [1-3]. Investigators in several studies evaluating goal-directed protocols have reported improved outcomes due to immediate treatment to prevent or resolve organ ischemia [4,5]. The PiCCO*plus *system (Pulsion Medical Systems, Munich, Germany) allows continuous CO measurement by pulse contour analysis (PCCO). Calibration of PCCO is performed by intermittent transcardiopulmonary thermodilution cardiac output (CO_TCP_). It has been demonstrated that PCCO agrees with pulmonary artery thermodilution CO [6-8] and with CO_TCP _[9,10] in cardiac surgery patients. However, the reliability of PCCO has been questioned in clinical scenarios such as acute hemorrhage and subsequent norepinephrine (NE) administration [11], changes in vascular tone [12], increased intra-abdominal pressure [13] or time interval between calibrations [14]. Therefore, the clinician needs to consider these confounders when interpreting PCCO values and prompting therapeutic decisions.

The present prospective observational study investigated a large group of critically ill patients with regard to whether agreement between PCCO and CO_TCP _is affected by different NE dosages or by the time interval between calibrations. On the basis of the existing literature, we generated the following two hypotheses: (1) Increasing NE dosage results in decreased agreement between PCCO and CO_TCP_, and (2) increasing the time interval between calibrations of PCCO results in decreased agreement between PCCO and CO_TCP_.

Only rare data are available about the usage of PCCO calibrations in clinical practice. Therefore, we retrospectively evaluated whether NE dosage or severity of disease as measured by the Acute Physiology and Chronic Health Evaluation II score (APACHE II score) had an influence on calibration frequency on our intensive care unit (ICU).

## Materials and methods

### Patients

In this prospective observational study, critically ill patients equipped with invasive hemodynamic monitoring by the PiCCO*plus *system (version 6.0) on our noncardiac ICU between September 2007 and July 2008 were included. The study was approved by our institutional review board in compliance with the Helsinki Declaration (Ethics Committee of the University Hospital Schleswig-Holstein, Campus Kiel, Kiel, Germany). Patients and/or relatives gave their informed consent for the patients' data to be used in the analysis. Invasive hemodynamic monitoring was performed according to the judgment of the attending physician on the ICU. Exclusion criteria were cardiac arrhythmias, a permanent pacemaker or any other mechanical cardiac support and known valvular heart disease.

### Hemodynamic measurements

In all patients, a central venous catheter and a thermistor-tipped arterial catheter (Pulsiocath; Pulsion Medical Systems, Munich, Germany) inserted via femoral artery were present upon enrollment. The PiCCO device uses pulse contour analysis according to a modified algorithm originally described by Wesseling *et al*. [15] to determine PCCO and is described in more detail elsewhere [9]. This algorithm enables continuous calculation of stroke volume (SV) by measuring the systolic portion of the aortic pressure waveform and dividing the area under the curve by the aortic compliance. Therefore, the PiCCO device needs to be calibrated by CO_TCP_. Calibrations were regularly performed by an ICU physician at defined time points (0:00 AM, 8:00 AM or 4:00 PM) with the patient in a supine position during a time period without acute hemodynamic instability using three subsequent boluses of 15 mL of ice-cold saline injected into the central venous line as proposed by the manufacturer [9]. During measurement, neither treatment provoking hemodynamic changes nor change of ventilation variables was performed. The dosage of vasopressors was kept constant. Our institutional guideline suggests calibration every 8 hours or before any major change in therapy is initiated. Therefore, additional calibrations by the attending ICU physician were allowed at any time. All hemodynamic data, including PCCO, central venous pressure (CVP), mean arterial blood pressure (MAP), pulse pressure (PP) (systolic minus diastolic aortic pressure) and heart rate (HR) were recorded immediately before and after calibration by CO_TCP_. Global end-diastolic volume index (GEDI) and systemic vascular resistance index (SVRI) were derived upon thermodilution. SV was calculated as CO_TCP _divided by heart rate. The PP to SV (PP/SV) relationship was used to examine the influence of NE dosage on central arterial stiffness as reported previously [16]. Our ICU is equipped with a patient data management system (PDMS) (CareSuite; Picis Inc., Wakefield, MA, USA) capable of electronically storing hemodynamic variables, including all single thermodilution calibrations, and ventilatory variables minute-by-minute.

### Statistical analysis

Statistical analysis was performed using the statistical software R (R Foundation, Vienna, Austria [17]) and GraphPad Prism 5.01 software (GraphPad Software Inc., San Diego, CA, USA). Data are reported as means ± standard deviations (SD) unless otherwise specified. NE subgroups were defined as no NE, low-dose NE (<0.1 μg/kg/min) and high-dose NE (≥0.1 μg/kg/min) according to the Sepsis-Related Organ Failure Assessment score [18]. Subgroups of time interval elapsed after the latest calibration were defined as <2 hours, 2 to 4 hours, 4 to 8 hours, 8 to 16 hours and 16 to 24 hours. Data subsets for hemodynamic variables, PP/SV ratio and calibration interval were compared using an unpaired two-tailed *t*-test. Comparison of PCCO and CO_TCP _was performed by using Bland-Altman statistics for multiple observations per individual [19], calculating mean differences between methods (bias) ±2 SD (limits of agreement). Bias between subgroups was compared using a *t*-test. The percentage error was calculated as reported by Critchley and Critchley [20], and interchangeability between methods was assumed as a percentage error below 30%. The precision of the reference technique (CO_TCP_) was analyzed according to the method described by Cecconi *et al*. [21] from the three consecutive bolus injections for calibration. To test whether PCCO reflected changes (Δ) in CO, the ΔPCCO (PCCO - preceding CO_TCP_) was analyzed against ΔCO_TCP _(actual CO_TCP _- preceding CO_TCP_) by linear regression analysis including the first pair of measurements of each patient. The influence of NE dosage and the severity of the patient's medical condition (APACHE II score) on calibration frequency was analyzed using the Spearman correlation for nonparametric data. *P *< 0.05 was considered statistically significant.

## Results

Seventy-three patients were included in this study. The median (interquartile range) APACHE II score of all patients was 24 (range, 20 to 29) at the time of inclusion. Detailed patient characteristics are given in Table 1.

**Table 1:** Patient characteristics, medical history and reason for instrumentation with PiCCO monitoring system^a^

Parameter	Value
Patients, *n*	73
Mean age, yr ± SD	63 ± 13; (range, 21 to 82)
Sex (males/females)	53/20
Weight, kg ± SD	79 ± 14
Height, cm ± SD	175 ± 8
APACHE II score	24 (range, 7 to 45)
Medical history, *n*	
None	6
Arterial hypertension	35
Chronic obstructive pulmonary disease	9
Coronary heart disease	7
Diabetes	12
Renal insufficiency	11
Reason for hemodynamic monitoring, *n*	
Hypovolemia (major surgery)	19
Hypovolemia (major trauma)	5
Peritonitis	15
Pneumonia	7
Resuscitated from cardiac arrest	5
Septic shock	22

^a^Data are means ± SD, absolute numbers or median (range). Multiple answers are possible. APACHE II score, Acute Physiology and Chronic Health Evaluation II score.

We obtained 330 data pairs. In 265 of 330 data pairs, patients received mechanical ventilation with a mean tidal volume of 8 ± 1 mL/kg, a mean fraction of inspired oxygen of 0.6 ± 0.1, a mean peak airway pressure of 23 ± 6 cmH_2_O and a mean positive end-expiratory pressure of 9 ± 3 cmH_2_O. In the remaining 65 data pairs, patients breathed spontaneously and received oxygen via face mask. Calibration interval was 9 ± 6 hours (range, 1 to 24 hours). The precision of the three bolus injection -CO_TCP _values was 7%, according to the method of Cecconi *et al*. [21].

Concerning the effect of NE dosage on the agreement between PCCO and CO_TCP_, 27 data pairs were excluded from further analysis because of additional dobutamine or epinephrine administration. In 161 of 303 data pairs, NE was administered in doses ranging from 0.01 to 4.29 μg/kg/min. The hemodynamic data and calibration intervals of different NE subgroups are presented in Table 2.

**Table 2:** Hemodynamic data and calibration interval of different norepinephrine subgroups^a^

	All	No NE	NE < 0.1 (μg/kg/min)	NE ≥ 0.1 (μg/kg/min)
Parameter	(*n *= 330)	(*n *= 142)	(*n *= 82)	(*n *= 79)
Hemodynamics				
CI (L/min·m^2^)	4.3 ± 1.1	4.4 ± 1.0	4.3 ± 1.0	4.3 ± 1.2
MAP (mmHg)	81 ± 15	88 ± 16	80 ± 11^b^	76 ± 13^b^
HR (beats/min)	98 ± 19	94 ± 16	96 ± 18	105 ± 21^b,c^
CVP (mmHg)	12 ± 5	11 ± 5	12 ± 5	13 ± 4
GEDI (mL/m^2^)	791 ± 191	808 ± 213	794 ± 180	780 ± 171
SVRI (dyn·s/cm^5^/m^2^)	1,367 ± 413	1,435 ± 409	1,309 ± 379	1,274 ± 419
Calibration interval (min)	443 (234 to 784)	442 (243 to 761)	518 (247 to 821)	439 (200 to 914)

^a^Data are given as means ± SD or medians (interquartile range); ^b^*P *< 0.05 vs. no NE; ^c^*P *< 0.05 vs. NE < 0.1. This table presents descriptive hemodynamic data and calibration interval regarding norepinephrine (NE) dosage subgroups. CI, cardiac index; MAP, mean arterial pressure; HR, heart rate; CVP, central venous pressure; GEDI, global end-diastolic volume index; SVRI, systemic vascular resistance index.

**Figure 1:**
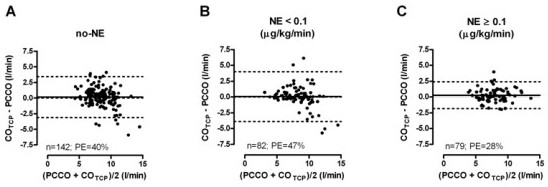
**Bland-Altman plots of different norepinephrine (NE) subgroups**. PCCO, pulse contour cardiac output; CO_TCP_, transcardiopulmonary thermodilution cardiac output; PE, percentage error; solid line, mean bias; dotted lines, limits of agreement.

Bias between NE subgroups did not differ significantly. However, PCCO was interchangeable with CO_TCP _only during high NE dosage and not at low or no NE dosage. The results of the Bland-Altman analysis are presented in Table 3, and plots are given in Figure 1.

**Table 3:** Results of Bland-Altman analysis of PCCO vs. CO_TCP_^a^

	Number of patients	Mean	Bias	Limits of agreement	Percentage error
Parameter	(*n*_all_/*n*_patient_)	(L/min)	(L/min)	(L/min)	(%)
All	330/73	8.1	0.16	-2.81-3.15	38
No NE	142/44	8.41	0.16	-3.12-3.44	40
NE < 0.1 (μg/kg/min)	82/38	8.50	0.06	-3.88-4.00	47
NE ≥ 0.1 (μg/kg/min)	79/30	7.87	0.29	-1.83-2.42	28^b^
Calibration interval 0 to 2 hours	36/25	8.00	0.25	-4.00-4.51	54
Calibration interval 2 to 4 hours	48/35	7.78	0.12	-3.37-3.60	46
Calibration interval 4 to 8 hours	95/41	8.21	0.09	-2.43-2.61	31
Calibration interval 8 to 16 hours	101/47	8.19	0.21	-3.17-3.59	42
Calibration interval 16 to 24 hours	50/28	8.06	0.23	-2.90-3.34	40

^a^*n*_all_, number of measurement pairs for pulse contour-derived cardiac output (PCCO) and transcardiopulmonary thermodilution cardiac output (CO_TCP_); *n*_patient_, number of patients; mean, mean of all PCCO and CO_TCP _measurements. ^b^Interchangeability according to Critchley and Critchley [20]. Bias and limits of agreement were calculated according to the method of Bland and Altman [19].

**Figure 2:**
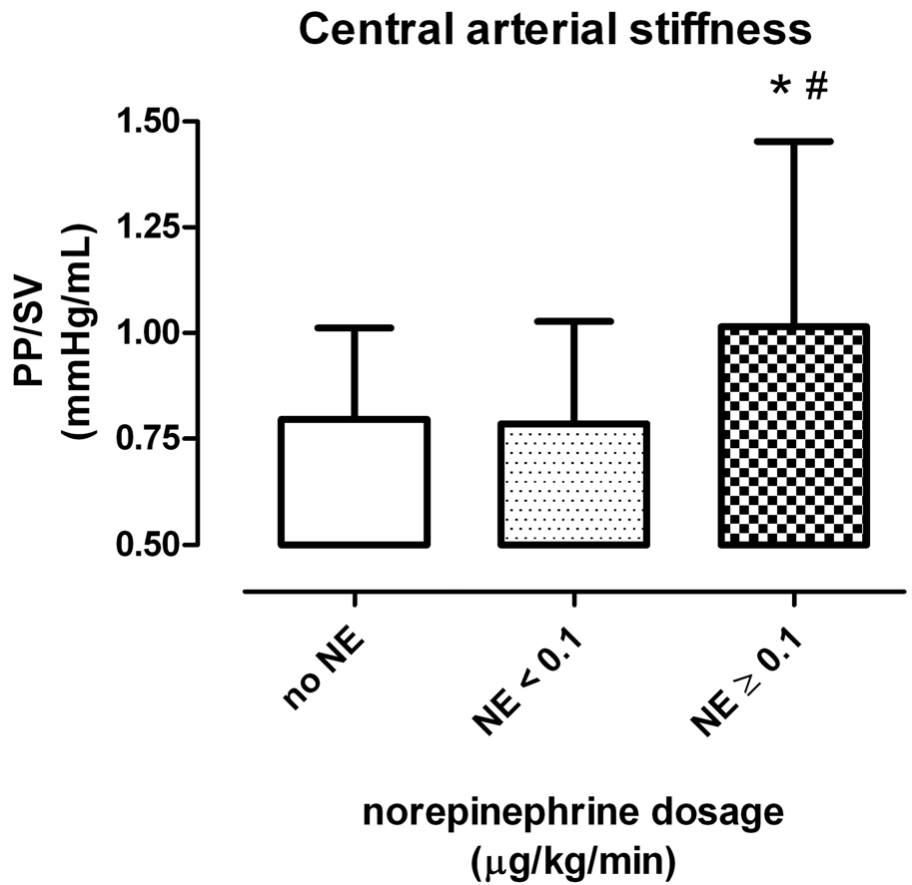
**Arterial stiffness**. Pulse pressure (PP) to stroke volume (SV) relationship (PP/SV) as a measure of central arterial stiffness within the different norepinephrine (NE) dosage (μg/kg/min) subsets. Data are means ± SD; *P < 0.05 vs. no NE; ^#^*P *< 0.05 vs. NE < 0.1 μg/kg/min.

The coefficient of correlation values, *r *(95% confidence interval (95% CI)), between ΔPCCO and ΔCO_TCP _was 0.46 (95% CI, 0.25 to 0.64; *P *< 0.001) for all patients, 0.19 (95% CI, -0.23 to 0.55; *P *= 0.36) for no NE, 0.37 (95% CI, -0.09 to 0.70; *P *= 0.11) for NE < 0.1 μg/kg/min and 0.78 (95% CI, 0.53 to 0.91; *P *< 0.001) for NE ≥ 0.1 μg/kg/min subgroups, respectively. In the NE ≥ 0.1 μg/kg/min subgroup, a statistically significant (*P *< 0.05) higher PP/SV relationship (arterial stiffness) was observed compared to the no NE or NE < 0.1 μg/kg/min subgroups, respectively (Figure 2).

**Figure 3:**
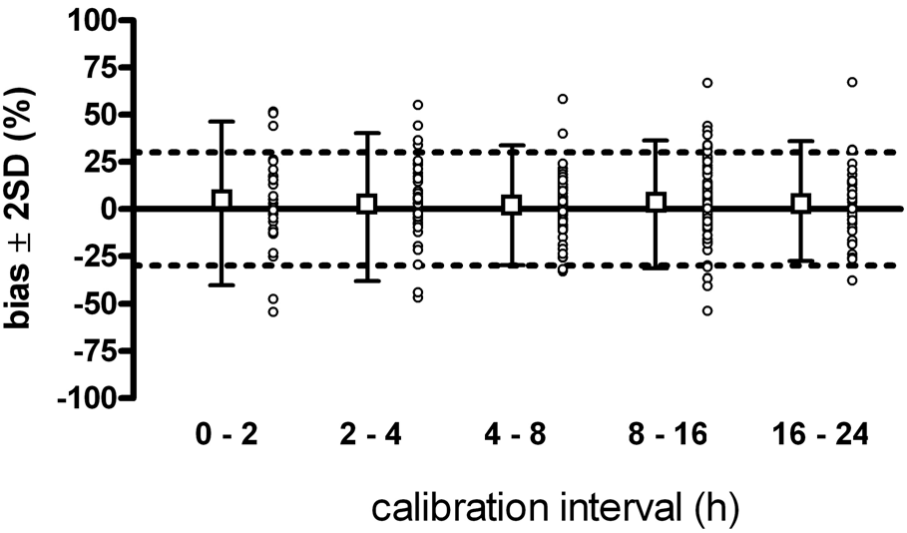
**Bias in relation to time interval between calibrations**. Mean bias (boxes) ± limits of agreement and individual bias (circles) expressed as percentage of CO_TCP _between PCCO and CO_TCP _in subsets of different calibration intervals. Dotted lines illustrate interchangeability (±30%).

The mean bias between PCCO and CO_TCP _did not depend on time elapsed from the preceding calibration. However, in none of the subgroups did agreement between PCCO and CO_TCP _meet defined criteria for interchangeability, as the percentage error was above 30% in all respective interval subgroups. The time-related effect on agreement is presented in Table 3. Individual bias during each interval, as well as mean bias ± limits of agreement, is plotted in Figure 3.

On our ICU, we recorded a mean (±SD) time interval after the preceding calibration of 9 ± 6 hours. In 151 (46%) recordings, the time interval exceeded the recommended 8-hour interval. In 14 (4%) recordings, the time interval was as long as 24 hours. The time interval did not correlate with NE dosage or APACHE II score (*r *= -0.04, *P *= 0.48; and *r *= -0.01, *P *= 0.41), respectively.

## Discussion

In the present study, we have demonstrated an influence of NE dosage on agreement of PCCO, as only during high NE dosage the criteria of interchangeability with CO_TCP _were met. Time elapsed between calibrations did not affect agreement between methods.

Goal-directed therapy in high-risk patients has been shown to improve outcomes [4,5]. One essential observation in these studies was that the earlier treatment was started, the better the outcome. Therefore, continuous CO monitoring in critically ill patients is needed. However, PCCO needs to be validated in a large number of patients and during relevant conditions to gain more insight into the mechanisms influencing this variable. The present study compared PCCO and CO_TCP _in 73 ICU patients with several comorbidities. Most previous studies compared PCCO with CO_TCP _in small series of patients during cardiac surgery [6,8,9,22]. Data from larger patient samples, however, are scarce. The percentage error between PCCO and CO derived by a thermodilution method varied between 26% and 50% in earlier studies [14,23]. Critchley and Critchley [20] defined a percentage error of less than 30% to indicate interchangeability. Accordingly, we found an acceptable agreement of PCCO with CO_TCP _only in data subsets obtained with high NE dosage, although a percentage error of 28% is still reasonably high. However, the results of the present study tend to refute our first hypothesis. Increasing NE dosage does not seem to be associated with decreased agreement between PCCO and CO_TCP_, but rather with improved interchangeability. PCCO further showed a better performance in tracking changes in CO during increased NE dosage because the coefficient of correlation between ΔPCCO and ΔCO_TCP _was higher. Vascular tone seems to be an important issue regarding the agreement of PCCO methods with a reference method such as transcardiopulmonary thermodilution. Rodig *et al*. [12] described an increased bias between PCCO and CO measured by thermodilution after administration of phenylephrine. The observed change of SVR >60% between calibrations may explain their findings. A recent publication applying the same PCCO software used in our study concluded that agreement was not influenced by changes in SVR due to better adaptation of the newer algorithm [14]. In the present study, SVR was not different between NE subgroups. Therefore, we hypothesize that despite a comparable SVR, a differing compliance of the vascular tree between subgroups of different NE dosages may explain the different level of agreement. A higher NE dosage may result in an increased central arterial stiffness and therefore reduced arterial compliance [24], as recently reported by Wittrock *et al*. [16]. In agreement with these findings, high NE dosage resulted in a significantly higher PP/SV relationship as an indicator of arterial stiffness. Increasing arterial stiffness leads to a more rigid vascular system and therefore may result in better agreement between methods. It is conceivable in this context that the vasculature of patients on high NE has less oscillatory capacity, which limits changes in arterial compliance and consequently on the deviation from the compliance obtained upon calibration. In clinical practice, however, many patients may be treated with either a low dose of NE or no NE, and according to our results, PCCO is not interchangeable with CO_TCP _in these patients.

Our results do not show a time-related effect on the agreement between PCCO and CO_TCP_, thus refuting the second hypothesis. The percentage error was above 30% in all calibration interval subgroups. The manufacturer recommends recalibration every 8 hours. Godje *et al*. [9] reported an overall acceptable agreement up to 44 hours; however, they did not indicate the bias and percentage error of subsets regarding different calibration intervals. Hamzaoui *et al*. [14] reported a percentage error below 30% only within the first hour after calibration of PCCO, but up to 37% within a 6-hour calibration interval. These authors concluded that PCCO is stable during a 1-hour period, and even changes in SVR did not alter the agreement. These results would prompt one to use hourly recalibration. Regarding our results, time elapsed from preceding calibration did not determine the level of agreement, as individually good agreement was observed up to 24 hours and individually poor agreement occurred within a period of 2 hours after calibration. Moreover, we found acceptable agreement in patients who were administered a high NE dosage, and thus had higher arterial stiffness, who had mean calibration periods of 7 hours.

This study also examined the clinical use of calibrations by using PiCCO technology. Our institutional guidelines recommend a recalibration of the PiCCO system every 8 hours (three times daily), as well as before and after any major change in therapy. We found that in only 54% of recordings were institutional guidelines of recalibration met. We did not observe a correlation of calibration frequency with APACHE II score or NE dosage, indicating that calibration of PCCO may not be dependent on the severity of critical illness. These findings are surprising, since recalibration may increase agreement between methods [13]. However, our results indicate that the time interval between calibrations may not to be the most important factor in determining PCCO accuracy; moreover, therapy during calibrations seems to be important.

There are some limitations to our study. To avoid additional risk due to a more invasive methodology of CO measurement, we used the PiCCO integrated transcardiopulmonary thermodilution instead of the pulmonary artery thermodilution method as a reference technique for PCCO as previously described [13,14]. The calibration interval was not strictly standardized to measure the effect of NE dosage on calibration frequency on our ICU.

## Conclusions

This study demonstrates further limitations of the PCCO method for the determination of continuous CO. Only during high NE dosage (≥0.1 μg/kg/min) was PCCO interchangeable with CO_TCP_. Therefore, the accuracy of PCCO measurement relies on important clinical circumstances.

## Key messages

• During clinical conditions, PCCO and CO_TCP _measurements cannot be used interchangeably in patients who are either not on vasopressor treatment or on a low dose of vasopressors.

• Acceptable agreement between the methods was observed only during an increased dose of norepinephrine, representing the minority of measurements. Even then the limits of agreement were rather large.

• The time interval between calibrations of PCCO does not improve the reliability of PCCO within a period of 24 hours.

## Abbreviations

Δ: delta, change in CO between actual and preceding calibration; APACHE II: Acute Physiology and Chronic Health Evaluation II score; CI: cardiac index; CO: cardiac output; CO_TCP_: transcardiopulmonary thermodilution cardiac output; CVP: central venous pressure; GEDI: global end-diastolic volume index; HR: heart rate; ICU: intensive care unit; MAP: mean arterial pressure; NE: norepinephrine; PCCO: pulse contour cardiac output; PE: percentage error; PP/SV: pulse pressure to stroke volume ratio; *r*: coefficient of correlation; SD: standard deviation; SV: stroke volume; SVRI: systemic vascular resistance index.

## Competing interests

BB is a member of the advisory board of Pulsion Medical Systems. MG, PM, JR, AC, OB, NW, JS and MS declare that they have no competing interests.

## Authors' contributions

MG conceived of the study design, carried out statistical analysis and drafted the manuscript. PM, OB and JR helped to draft the manuscript. AC supported statistical analysis. NW, JS and MS coordinated the study. BB conceived of the study design, coordinated the study and helped with statistical analysis and drafting of the manuscript. All authors read and approved the final manuscript.
